# When Significant Others Suffer: German Validation of the Burden Assessment Scale (BAS)

**DOI:** 10.1371/journal.pone.0163101

**Published:** 2016-10-20

**Authors:** Christina Hunger, Lena Krause, Rebecca Hilzinger, Beate Ditzen, Jochen Schweitzer

**Affiliations:** 1 Institute of Medical Psychology, Center for Psychosocial Medicine, University Hospital Heidelberg, Bergheimer Straße 20, 69115 Heidelberg, Germany; 2 Institute of Psychology, University of Heidelberg, Hauptstraße 47-51, 69115 Heidelberg, Germany; IRCCS Istituto Auxologico Italiano, ITALY

## Abstract

There is a need of an economical, reliable, and valid instrument in the German-speaking countries to measure the burden of relatives who care for mentally ill persons. We translated the Burden Assessment Scale (BAS) and conducted a study investigating factor structure, psychometric quality and predictive validity. We used confirmative factor analyses (CFA, maximum-likelihood method) to examine the dimensionality of the German BAS in a sample of 215 relatives (72% women; *M* = 32 years, *SD* = 14, range: 18 to 77; 39% employed) of mentally ill persons (50% (ex-)partner or (best) friend; *M* = 32 years, *SD* = 13, range 8 to 64; main complaints were depression and/or anxiety). Cronbach’s *α* determined the internal consistency. We examined predictive validity using regression analyses including the BAS and validated scales of social systems functioning (Experience In Social Systems Questionnaire, EXIS.pers, EXIS.org) and psychopathology (Brief Symptom Inventory, BSI). Variables that might have influenced the dependent variables (e.g. age, gender, education, employment and civil status) were controlled by their introduction in the first step, and the BAS in the second step of the regression analyses. A model with four correlated factors *(Disrupted Activities*, *Personal Distress*, *Time Perspective*, *Guilt)* showed the best fit. With respect to the number of items included, the internal consistency was very good. The modified German BAS predicted relatives’ social systems functioning and psychopathology. The economical design makes the 19-item BAS promising for practice-oriented research, and for studies under time constraints. Strength, limitations and future directions are discussed.

## Background

The image of psychiatric and psychotherapeutic care in Germany has changed fundamentally over the past four decades. This change has been accompanied by a continuous decrease in the number of beds in hospitals and a shortening of the duration of [1, 2]. Consequently, care duties and responsibilities now are transferred particularly to the families of the mentally ill [1, 3]. This is followed by a multitude of caregiver burden [4, 5]. Perlick and colleagues [6] found 82% of 500 relatives of people with bipolar disorder to be impaired, while 7% reported severe burden. Prevalence rates for some forms of stress vary widely between 55% and 90%. Most studies on caregivers focus on the quantity of burden (i.e. how much burden), the quality of burden (i.e. which factors emerge in content and statistical analyses), the coping with difficult situations, and screening tools to measure distress and coping strategies [4]. Hoenig and Hamilton [7] were the first who differentiated between objective and subjective stress factors: objective stress factors refer to observable changes like financial difficulties, limitations in leisure time, health problems, disadvantages at work or negative impact on interpersonal relationships; subjective stressors include the experience of helplessness, guilt, grief, rejection, or fear of the future [8]. Reviews about the most commonly used instruments to assess caregiver burden show that there is still no standard tool for detecting distress of caregivers due to their conceptual heterogeneity, different definitions of “distress”, their often limited psychometric quality, and their concentration on relatives of schizophrenic patients [9, 10]. Additionally, most instruments are time-consuming or require special interview training [11].

The detection of relatives’ distress however is important for two reasons: (1) relatives are at increased risk of mental diseases, thus require more treatment and medical care [12–14]. Prevention and treatment providers are in need of a better understanding of caregivers’ mental and physical impairment if they aim at a sustainable support for both the mentally ill and the also often impaired relatives [15]. (2) The detection of changes in family members’ burden is an important outcome considering the evaluation of social systems interventions [16]. Social systems are understood as a collectivity of individuals who are bound together as a unit by multiple interactions [17] and differentiated from their environment by a boundary of meaning [16]. In the case of mental illness, both the affected individual as well as the members of the social system it belong to may seek help. However, little is known about how caregivers benefit from psychotherapeutic interventions, independently from being involved or not.

As far as known by the authors, three instruments exist that can be used to examine relatives’ distress in German-speaking countries: the German version of the Questionnaire for Family Problems [18], the German version of the Involvement Evaluation Questionnaires [19] and the German Questionnaire about Relatives Distress [20]. However, these instruments still miss an evaluation of the factor structure [19], reliability [20], or they are limited to schizophrenic patients [18].

### Description of the Burden Assessment Scale (BAS)

The Burden Assessment Scale (BAS) [11] measures objective as well as subjective distress of relatives or intimate others within the past six months. In contrast to similar instruments, its 19 items focus on persistent stress experiences [9]. The BAS is not limited to a specific group (e.g. relatives of schizophrenic patients [18]), but can be used with any relative supporting a person with a severe mental disorder. The BAS demonstrated validity by relatives seeking psychotherapeutic support and who attained higher scores of distress on the total BAS compared to those who did not search for professional support. Convergent validity was shown by correlations with the Perceived Family Burden Scale [21], the Zarit Caregiver Burden Scale [22] and the Grief Scale [23, 24]. Predictive validity was not analyzed in these studies.

Two original studies, the New Jersey Division of Mental Health and Hospitals study (DMH&H) and “The Club” study demonstrated best fit for the BAS on a model with five independent factors [11]. The factors, however, did not cover the same items. Based on the 94 relatives included in the DMH&H study, (1) the first factor *Disrupted Activities* encompassed distress of relatives resulting from difficulties in concentrating, altered personal plans, limitation in leisure time activities, changed habits in the household, reduced time for friends and neglect of needs from other family members; (2) the second factor *Personal Distress* referred to frictions in the relatives’ surrounding, feelings of shame, stress caused by too many demands or the feeling of being trapped in the supporting role; (3) the third factor *Time Perspective* summarized the caregivers’ sorrows with respect to the dealing with the mentally ill’s disorder over time, including the relatives’ insight of a changed intimate person and the suffering from the stigma of the disorder; (4) the fourth factor *Guilt* asked for feelings of guilt caused by assistance of little value or thoughts of having caused the mentally ill’s problems; (5) the fifth factor *Basic Social Functioning* involved items about missing days at work/school and family frictions. „The Club”study included 94 caregivers and revealed the same factors, except for the fifth factor. The covering of the items slightly differed, and the fifth factor was labeled *Worry* encompassing difficulties to concentrate as well as relatives’ concern to have not provided enough help.

In both the DMH&H and “The Club” study, objective factors *(Disrupted Activities*, *Basic Social Functioning)* and subjective factors (*Personal Distress*, *Time Perspective*, *Guilt)* could be separated, while *Worry* contained items of both the objective and subjective dimensions. In both studies, the five-factor model explained 66% of the variance, while *Disrupted Activities* explained most of the variance (40%, DMH&H; 37%, “The Club”). Internal consistencies revealed very good (Cronbach’s *α* .89 to .91) [11]. International studies including US American, Canadian, Puerto Rican, Swedish, Taiwanese, Turkish, Australian, and African-American samples replicated these finding [24–30], but did not investigated the predictive validity of the BAS.

Altogether, it can be said that the BAS covers a broad spectrum of relatives’ distress, and that it appears fruitful for both research and clinical practice due to its economy, very good internal consistencies and demonstrated validity [11]. This makes the BAS worth to be considered a promising instrument for the assessment of caregiver burden in German-speaking countries. However, we still miss its validation in a German—speaking sample.

### Aim

This study aimed at investigating the factor structure, psychometric quality and predictive validity of the German BAS in relatives of psychotherapy patients. We expected a model with five independent factors *(Disrupted Activities*, *Personal Distress*, *Time Perspective*, *Guilt*, *Basic Social Functioning or Worry)* showing best fit according to the original BAS [11]. Validation measures were hard to find since the existing German instruments concentrated on relatives of schizophrenic, cancer or dementia patients, or still miss psychometric validation. They did not appear to be appropriate for relatives of psychotherapy patients in the broader sense. However, dementia research demonstrated that relatives experience less distress when they perceive a positive relationship to the intimate ill [28, 31]. Reduced caregiver burden was associated with a stable social network and positive social support [32]. To investigate the prediction of social systems functioning by caregiver burden, we used the Experience in Social Systems Questionnaire (EXIS) [33–35] that measures the individual’s feelings of belonging, autonomy, accord and confidence within its important personal and organizational social systems (EXIS.pers, EXIS.org). Additionally, the more burdened the relatives the more often they report psychosomatic pathology [12, 14, 36]. To investigate the prediction of psychopathology by caregiver burden, we also used the Brief Symptom Inventory (BSI) [37] that measures individuals’ physical and mental symptoms. Altogether, we expected the prediction of caregivers experience in their social systems and their psychopathology by caregiver burden: relatives with much burden should describe less positive experience within their important personal and organizational social system, and they should report more psychosomatic complaints.

## Method

### Translation

According to the guidelines of the European Social Survey Program [38], CH, LK and four bilinguals from the Institute of Medical Psychology independently translated the BAS. Difficulties were discussed with the authors of the BAS [11] to ensure connotative and text-normative equivalence of the English and German items and instructions [39], and comprehensibility for German-speaking countries. An additional independent bilingual subsequently examined the first German version of the BAS. As proposed by Schmitt and Eid [40], seven bilinguals worked on a back translation. Minor item-revisions were incorporated into the second German version of the BAS. Finally, three German-speaking test readers confirmed its good comprehensibility.

The German BAS differs from the original by using the introductory sentence *Because of the mental illness of the person in therapy*, *I did/I was…* instead of the introductory question *Because of (name’s) illness*, *to what extend have you…*?. This adjustment was made since the BAS centers an individual perspective. In German-speaking countries, this is more strongly supported by the first-person perspective in contrast to the interrogative form.

### Procedure of the study

Based on a cross-sectional design, our study “Experience of Interpersonal Relationships” run online using the survey software UNIPARK for 138 days between December 2014 and May 2015. Interested persons initially were informed about the study aims, eligibility requirements and data-protection. If they wanted to participate, they provided their written consent and generated a personal code. They then filled in their demographic data and those of the mentally ill, in addition to the BAS, EXIS.pers, EXIS.org, and BSI. Finally, the participants had the possibility to take part in a drawing of three Amazon vouchers worth 50€ each.

### Recruitment and inclusion/exclusion criteria

Participants were recruited via flyers and announcements in hospital newsletters, online magazines, Facebook groups and internet forums, by addressing therapists, psychotherapy institutions and self-help groups for relatives, and by the distribution of announcements using the mailing lists of various German student councils.

Inclusion criteria required participants (1) to be close to someone with a mental disorder currently in inpatient or outpatient psychotherapy, (2) to be at least 18 years old, and (3) to agree to participate in the study. Exclusion criteria referred to violations of the inclusion criteria.

### Measures

**Fig 1:**
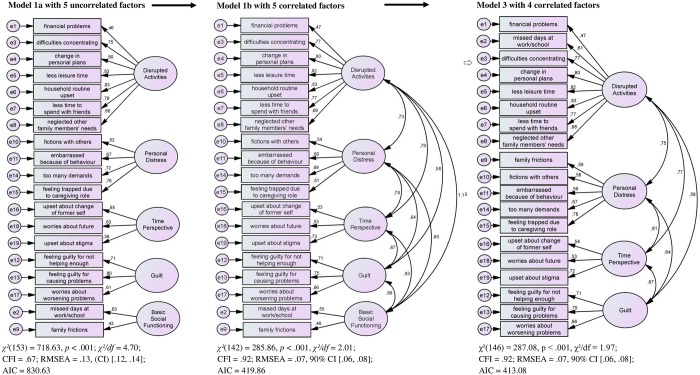
Structural Equation Models (Confirmatory Factor Analyses, CFA; Maximum-Likelihood Estimation). Model 1a is the original DMH&H study, five uncorrelated factors. Model 1b is the original DMH&H study, five correlated factors. Model 3 is the modified BAS model, four correlated factors.

**Fig 2:**
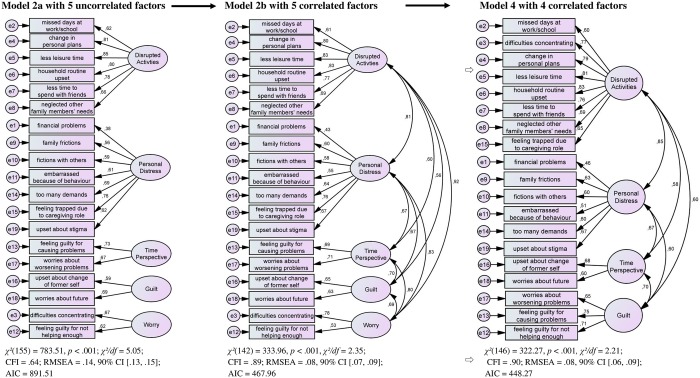
Structural Equation Models (Confirmatory Factor Analyses, CFA; Maximum-Likelihood Estimation). Model 2a is the original DMH&H study, five uncorrelated factors. Model 2b is the original DMH&H study, five correlated factors. Model 4 is the modified BAS model, four correlated factors.

The 19-item **Burden Assessment Scale (BAS)** serves the assessment of caregivers’ objective and subjective burden within the past six months. Answers are given on a 4-point Likert scale ranging from (1) *not at all* to (4) *very much*, with higher values indicating stronger burden. The original studies and factor structure of the BAS is described above, item-specific content is shown in Figs 1 and 2. The original BAS shows a very good internal consistency with Cronbach’s *α* = .91 in both the DMH&H and “The Club” study [11].

The 12-item **Experience in Social Systems Questionnaire (EXIS.pers, EXIS.org)** [33–35] assesses individuals’ experience within their important personal (e.g. family, friends) and organizational (e.g. teams, work units) social systems within the past two weeks, including four dimensions *(belonging*, *autonomy*, *accord*, *confidence)*, on a 6-point Likert scale from (1) *not at all* to (6) *entirely*. High values indicate strong and positive experience in one’s important social systems. The EXIS shows very good to satisfactory internal consistencies, validity is demonstrated with interpersonal, psychotherapeutic, social support and work-related constructs, and sensitivity to change after 3-day Family Constellation Seminars [34].

The 53-item **Brief Symptom Inventory (BSI)** [37] assesses a variety of physical and mental symptoms based on a *global severity index (GSI)* and nine subscales (*somatization*, *obsessive-compulsive*, *interpersonal sensitivity*, *depression*, *anxiety*, *hostility*, *phobic anxiety*, *paranoid ideation*, *psychoticism*) within the past seven days on a 5-point Likert scale from (0) *not at all* to (4) *very strong*. High values indicate strong impairment. The BSI demonstrates high convergent validity to the SCL-90-R [41]. It has been used in studies with relatives of patients with a borderline personality disorder [42], affective disorder [43] and schizophrenia [44]. The BSI-GSI shows excellent internal consistency with Cronbach’s *α* = .92 [37].

### Statistical Design

All analyses were calculated using the statistical packages SPSS (Version 20.0, IBM Germany) and AMOS [45]. Using *confirmatory factor analysis* (CFA, maximum-likelihood estimation), we tested the five-factor models of both original studies, the DMH&H study and “The Club” study [11] (Fig 1, model 1a; Fig 2, model 2a). We set variance of latent factors to 1 in both models, and equaled factor loadings within the factors for those which included less than three items (model 1a: *Basic Social Functioning*; model 2a: *Time Perspective*, *Guilt*, *Worry*). Since the chi-square test easily becomes significant due to its dependence on the sample size, the indices CFI, RMSEA and AIC acted as indicators of the model fit [46]. Hu and Bentler [47] recommend a cutoff score of CFI ≥ .95 for a very good fit of the data to the model. RMSEA scores ≤ .05 indicate a good model fit, scores between .06 and .08 are acceptable, and scores ≥ .10 indicate a weak model fit [46]. AIC [48] was used to detect the best model indicated by the lowest AIC. Cronbach’s *α* served to assess the *internal consistency*. According to Bühner [49], α > .90 indicates very good, .80 < α < .89 good, and .70 < α < .79 satisfactory internal consistencies [50]. Due to the high sensitivity of *α* to the number of items included, we additionally calculated confidence intervals (CIs) for Cronbach’s *α* [51].

We used regression analyses to test the *predictive validity*, i.e. the prediction of relatives’ experience within their personal (EXIS.pers) and organizational (EXIS.org) social systems as well as their degree of psychopathology (BSI-GSI) by their burden (BAS). Variables that might have influenced the dependent variables (e.g. age, gender, education, employment and civil status) were controlled by their introduction in the first step, and the BAS in the second step of the regression analyses.

## Results

### Sample

In sum, 238 relatives filled in the BAS and additional questionnaires. Of these, 23 participants showed missing values of more than 20%, and were deleted from further analyses. The final BAS sample included 215 relatives: 72% were women (*M* = 32 years, *SD* = 14, range: 18 to 77), 64% lived with a partner, 86% had a college degree, 39% were employed and 51% studied, 93% were Germans and 97% spoke German as their mother tongue. Most study participants referred to their (ex-)partner (28%), a (best) friend (26%), a parent (17%), sibling (14%) or child (11%) when answering the BAS. Those in psychotherapy had almost the same mean age as their caregiver though the range of years also included children and adolescents (*M* = 32 years, *SD* = 13, range 8 to 64). Relatives described the following disorders with respect to the mentally ill, while multiple ratings were allowed: depression (65%), uncertainty in social interactions (37%), anxiety (30%), obsessiveness (8%), aggressiveness (7%), phobias (7%), paranoia (7%), psychoticism (5%), and others (8%). According to the caregivers, the mentally ill participated in behavioral, psychoanalytic or psychodynamic psychotherapy (57%), systemic psychotherapy (10%), or the relative did not know the school the intimate other was treated with (33%).

### Confirmatory factor analysis

Both model 1a with five uncorrelated factors according to the DMH&H study (Fig 1) and model 2a with five uncorrelated factors according to “The Club” study (Fig 2) showed poor fit to the data. Due to substantial correlations between the factors, it could be assumed that the German BAS factors assess similar aspects of caregiver burden. We tested this assumption using the original models from the DMH&H study and “The Club” study, while allowing the five factors in each model to be correlated (Fig 1, model 1b; Fig 2, model 2b). The results supported our assumption.

To obtain a better and more economical model, and due to the finding that the fifth factor with only two items showed highest inter-correlations with other factors, we reallocated its items according to their second highest factor loading. In the modified model 3 of the DMH&H study (Fig 1), *Disrupted Activities* received item 2 (*Because of (name’s) illness*, *[I] missed days at work/school*), and *Personal Distress* received Item 9 (*Because of (name’s) illness*, *[I] experienced family frictions and arguments*). In the modified model 4 of “The Club” study (Fig 2), *Disrupted Activities* received Item 3 (*Because of (name’s) illness*, *[I] found it difficult to concentrate on [my] own activities*), and *Guilt* received Item 12 (*Because of (name’s) illness*, *[I] felt guilty because [I was] not doing enough to help*). In a subsequent CFA, we tested both modified model 3 and 4, each with four correlated factors. Both models showed a better fit to the data. More precisely, model 3 with four correlated factors fitted slightly better because of the higher CFI, lower RMSEA and AIC.

In conclusion, the structural equation model 3 with the four dependent variables *Disrupted Activities* (items 1 to 8), *Personal Distress* (items 9 to 11, 14, 15), *Time Perspective* (items 16, 18, 19) and *Guilt* (items 12, 13, 17) provides the best and clearest results in our study with a German-speaking sample. We thus will call this model the modified German BAS. Our findings contradict our initial hypothesis that a model with five independent variables reveals the best fit, as it was demonstrated in both the original DMH&H and “The Club” study [11].

### Descriptive statistics

Mean score for the BAS total score was 2.29 (*SD* = 0.66), and subscales ranged from 2.11 to 2.73 (*SD* = 0.77 to 0.91) (Table 1).

**Table 1:** Descriptive Statistics and Internal Consistencies.

Scale	*M*	*SD*	*T*-value	*SE*	*α*	CIs[LC; UC]
**BAS Total Score**	2.29	0.66		0.05	.92	[.90; .93]
Disrupted Activities	2.11	0.77		0.05	.90	[.88; .92]
Personal Distress	2.20	0.79		0.05	.78	[.73; .82]
Time Perspective	2.73	0.83		0.06	.74	[.67; .79]
Guilt	2.39	0.91		0.06	.64	[.55; .72]
**EXIS.pers Total Score**	4.02	0.98		0.07	.94	[.93; .95]
**EXIS.org Total Score**	4.04	1.02		0.08	.95	[.94; .96]
**BSI-GSI**	0.61	0.49	63	0.03	.96	[.95; .97]
Somatization	0.47	0.47	58	0.03	.70	[.63; .76]
Obsessive-Compulsive	0.83	0.61	58	0.04	.70	[.63; .76]
Interpersonal Sensitivity	0.56	0.64	56	0.04	.72	[.65; .78]
Depression	0.49	0.52	58	0.04	.71	[.65; .77]
Anxiety	0.73	0.61	61	0.04	.73	[.67; .78]
Hostility	0.81	0.73	63	0.05	.74	[.68; .79]
Phobic Anxiety	0.39	0.43	61	0.03	.54	[.43; .63]
Paranoid Ideation	0.66	0.66	60	0.05	.68	[.60; .74]
Psychoticism	0.69	0.58	65	0.04	.66	[.58; .73]

*Note*. BAS-GSI = Burden Assessment Scale, Global Severity Index; EXIS.pers = Experience In Personal Social Systems; EXIS.org = Experience In Organizational Social Systems; BSI = Brief Symptom Inventory.

### Internal Consistencies

Cronbach’s *α* for the BAS total score was .92 (CI: .90 to .93), and ranged from .64 to .90 for the subscales (CIs: .55 to .92). The EXIS.pers total score showed *α* at .94 (CI: .93 to .95), and the EXIS.org total score showed *α* at .95 (CI: .94 to .96). The BSI-GSI showed *α* at .96 (CI: .95 to .97), and ranged from .54 to .74 for the subscales (CIs: .43 to .79) (Table 1).

### Predictive Validity

The total variance of caregivers’ experience in their personal and organizational social systems explained by the whole model was about 15% and 9%, respectively. For the prediction of relatives’ mental and physical complaints, the total variance explained by the whole model was about 10%. Relatives’ burden alone significantly accounted for about 14%, 9%, and 10%, respectively, over and above the variance explained by the demographic data. Neither age, gender, education, civil status, nor employment revealed significance. The only exception was made with respect to the prediction of the caregivers’ better experience in their personal social system when being employed. As expected, the regression analyses indicated that the higher the relatives’ burden the less positive was the experience in their personal and organizational social systems, and the higher were their psychosomatic complaints (Table 2).

**Table 2:** Hierarchical regression analysis: Relatives’ burden predicting their experience in personal and organizational social systems and psychopathology (n = 215).

Variables entered	R	R^2^_corr_	ΔR^2^	B	SE B	*β*	*t*
**Prediction of relatives’ experience in their personal social systems (EXIS.pers)**							
Step 1	.191	.012	.036				
Age				.006	.006	.084	.965
Gender				-.012	.142	-.006	-.085
Education				.162	.094	.120	1.723
Civil status				.007	.080	.007	.088
Employment				.064	.024	.187	2.630**
Step 2	.408	.141	.130***				
Relatives‘ burden				-.558	.100	-.377	-5.576***
**Prediction of relatives’ experience in their organizational social systems (EXIS.org)**							
Step 1	.178	.003	.032				
Age				.016	.008	.200	1.931
Gender				.048	.163	.022	.296
Education				.136	.119	.086	1.136
Civil status				.011	.104	.009	.103
Employment				.038	.030	.106	1.276
Step 2	.349	.090	.090***				
Relatives‘ burden				-.496	.119	-.313	-4.167***
**Prediction of relatives’ psychopathology (BSI-GSI)**							
Step 1	.156	.000	.024				
Age				-.003	.003	-.071	-.780
Gender				.002	.074	.001	.022
Education				-.075	.049	-.110	-1.519
Civil status				-.046	.042	-.096	-1.104
Employment				.000	.013	-.002	-.027
Step 2	.313	.098	.070***				
Relatives‘ burden				.210	.052	.283	4.018***

*Note*. BAS = Burden Assessment Scale, total score; EXIS.pers = Experience In Personal Social Systems, total score; EXIS.org = Experience In Organizational Social Systems, total score; BSI-GSI = Brief Symptom Inventory, total score. The reported B, SE B, *β* and *t*-test values in the table are the results of the final regression model including all predictors in the two steps.

*p ≤ .05;

**p ≤ .01;

***p ≤ .001

## Discussion

This study describes the first German validation of the BAS [11] in a sample of 215 relatives of mentally ill persons being in psychotherapeutic treatment. We found a four-factor structure with correlated factors (*Disrupted Activities*, *Personal Distress*, *Time Perspective*, *Guilt)* showing the best model fit (Fig 1, model 3). The internal consistency for the BAS total score was very good, with a small confidence interval considering Cronbach’s *α*. The BAS predicted caregivers’ experience in their personal and organizational social systems (EXIS.pers and EXIS.org) as well as the degree of their psychosomatic complaints (BSI). Though the goal of our study was not to test objective and subjective burden as separated constructs, our analyses may support the assumption that they describe distinctive aspects. The modified German BAS with its 19 items appears economical and may serve the administration in future research and daily practice.

Considering the degree of relatives’ burden in our study, we found it equal to other studies including the BAS and populations like relatives of patients with anxiety, depression, schizophrenia or other severe mental disorders [11, 26–30, 52, 53]. Relatives worried mostly about the future of the intimate other being in psychotherapy which supported the findings from the original US American samples [11], Afroamerican, Schwedish and Australian samples [26, 27, 30]. In contrast, in our German study, financial problems, missing work/ school, frictions with others and guilt feelings caused less burden compared to findings from these international studies. This may have been caused by national differences in the social safety or health networks. For example, Germany is well-known for its very supportive social insurance system which may has functioned as a buffer.

In contrast to the original study [11], we found a four-factor model with correlated factors demonstrating best fit. The items of the original fifth factor in the DMH&H study as well as “The Club” were reallocated according to their second highest factor loading. In the modified German BAS (Fig 1, model 3), *Disrupted Activities* also included “missed days at work/school”, and Personal Distress encompassed the experience of “family frictions and arguments”. Other studies also showed a four-factor model with the best fit [29, 30]. In a Canadian sample, Murdoch and colleagues [29] found *Role Restriction*, *Family Impact*, *Public Embarrassment* and *Guilt/Worry*. *Role Restriction* complies to a great extent with *Disrupted Activities*, and *Guilt/Worry* resembles *Guilt* when compared to our German study. The allocation of items to factors still remains difficult. In an Australian sample, Page and colleagues [30] labelled the factors in accordance with the original study [11], but included different items per factor. It is crucial to consider this different item composition per factor, above all when interpretations will be made on the subscale level and across distinct population. Divergence in item composition impede the precise distinction of variation in outcome attributable to the BAS as a measure of caregiver burden versus the different perception and/or reporting of burden as possibly a culture bound construct [26, 49]. Consequently, future studies using the BAS should rely on the total score and discuss their limited generalizability with respect to the studied populations.

Considering the content-related meaning of the four-factor structure, we draw on Guada and colleagues [26] who used the BAS in an African-American sample with low socioeconomic status. They found two factors of which one assesses objective and the other one subjective burden. *Personal Distress*, *Time Perspective* and *Guilt* did not emerge as specific factors. The authors assumed that low-income African-American relatives experience “burden as one major or broad component in their lives similar to other areas that demand ongoing coping and adaptation” (p. 233) whereas Americans like in the original study [11] may have reported and/or experienced “a more personalized form of subjective caregiver burden” (p. 239) [11]. The authors did not discuss whether the reported differences are due to either ethnic affiliation or socioeconomic status. Unfortunately, we did not ask for socioeconomic status in our study. We assume that we have recruited well-educated relatives with good socio-economic status. Our assumption grounds in the recruitment of an internet sample, while online studies found to often been composed by participants at the high end of the socio-economic and educational spectrum [54]. In this sense, our German sample seems to be similar to the original sample of the BAS including US Americans [11].

Another difference to the original study [11] refers to the allowance for the factors to be correlated. Unfortunately, the authors of the original BAS did not explain their premise of uncorrelated factors. Inter-correlations between *Disrupted Activities*, *Personal Distress*, *Time Perspective* and *Guilt* are in accordance to theoretical and empirical considerations of burden. Likewise in our study, the Australian BAS included inter-correlated factors [30]. Objective and subjective factors often correlate [55, 56]. Alternative questionnaires of burden also considered factors to be correlated [57–59]. The separation of objective and subjective factors thus has some limits. In the original BAS, item 1 to 10 described objective and item 11 to 19 subjective burden. In our study, *Disrupted Activities* pictured objective burden, *Time Perspective* and *Guilt* subjective burden, and *Personal Distress* included both items of objective and subjective burden. These findings support results from BAS studies which applied the criteria of the original BAS [11], but did not demonstrate objective and subjective burden to be independent factors [25–27, 29, 30]. In our opinion, the existence of correlated factors may be explained by difficulties to answer the BAS items exclusively from either the objective *or* subjective perspective, while the BAS does not offer different instructions for both perspectives. For example, one may think of family frictions in terms of their frequency (objective) *and* intensity (subjective). Possibly, it would be worthwhile to split the BAS into two domains with different instructions. The Family Burden Questionnaire [20] may serve as an example: relatives can first specify whether or not they have made a specific experience, and if so, they can estimate the intensity of burden related to this experience.

Considering the internal consistencies of the BAS, the total score showed a very good internal consistency, with a small confidence interval. Alpha statistics are highly sensitive to the number of items included [51]. The BAS contains 19 items, thus we could have expected a high internal consistency. We however know other scales that lack good internal consistencies though the number of items is similar to the full BAS [60]. Consequently, it was not evident that the total score of the BAS revealed such good internal consistency. The *Disrupted Activities* factor showed an internal consistency that was as high as the one for the total score. It would be worthwhile to think about the shortening of the BAS in further studies with respect to items that, for example, encompass distress of relatives resulting from difficulties in concentrating, altered personal plans, changed habits in the household or reduced time for friends. Such procedure would support the economy of the BAS. The *Time Perspective*, in contrast, may profit from some additional content-related items. The internal consistency was satisfactory, while the factor contained no more than three items. The number of three items represents the minimal requirement for factor composition which often comes along with low internal consistencies [51]. Consequently, reliability may improve with two or three items that measure relatives’ dealing with the mentally ill’s disorder over time.

A further aim of our study was the predictive validation of the German BAS using the EXIS.pers, EXIS.org and BSI. Predictions were in the hypothesized direction, indicating that the higher the caregiver burden the less positive their experience in their personal and organizational social systems, and the higher their report of psychosomatic complaints. Consequently, relatives with much burden experience less feelings of belonging, autonomy, confidence and accord within their social system, be it a personal or organizational social system. Additionally, caregivers with much burden indicate stronger psychosomatic complaints. Though predictive validity was not analyzed in former studies on the BAS, our findings support those investigations which showed associations of the BAS with other scales measuring family and caregiver burden as well as psychosomatic symptoms [21–24]. It would be worthwhile to assess the BAS in future intervention studies to assess their sensitivity to change and to become able to reliably and validly assess change of relatives’ burden in association with patients’ therapeutic change.

The explained variance of relatives’ experience in their social systems and their psychopathology by the experienced burden is not highly substantial. This may have been caused by how the caregivers composed their social systems while answering the questionnaires. Both EXIS.pers and EXIS.org allow the respondents to include all people which they consider to be important members of their social system. According to our findings in the original EXIS.pers study, people think in combinations of social system members: partner and children; partner and friends; parents, partner and friends; parents, children and others [33]. The relatives in our study may have thought of more persons than the mentally ill only. They may have also included those who assist them when giving care, which may have resulted in a reduction of explained variance. Additionally, the EXIS.pers and EXIS.org do not require the person in psychotherapy to be included in the relative’s important social system. This may have caused additional variation in the composition of the social system. Future studies should introduce the EXIS.pers and EXIS.org with a more specific focus. They also should include alternative instruments which better relate to the construct of burden. For German-speaking countries, however, we are still faced with the lack of appropriate instruments for relatives of patients with different mental and physical disorders. In contrast, the majority of the existing instruments addresses caregivers of patients with specific severe mental or physical disorders like schizophrenia or cancer, or still are in need of psychometric validation.

Considering the small amount of variance in relatives’ psychopathology explained by the burden they felt while caring for the mentally ill person, it again needs to be emphasized that the person in psychotherapy did not need to suffer from acute and/or severe impairment in our study. We recruited relatives of patients in psychotherapy in general. Consequently, we assumed that the prediction of relatives’ psychosomatic complaints by their burden will be stronger if they care for patients with acute and severe mental impairment.

### Strengths, Limitations, Future Directions

The major strength of the modified German BAS refers to its provision of first evidence to its psychometrical soundness grounding in conservative analyses. This makes the BAS available for further investigations in German-speaking countries. We recommend using the BAS total score for investigations in the near future until we have a clearer picture of the factor structure of the German BAS. The economical design makes the BAS promising for practice oriented research [61], and for studies under time constraints. The major limitations refer to our small sample size and the non-representative sample when it comes to drawing clinically relevant and generalized conclusions for caregiver burden. Future studies should examine the BAS with a representative sample in order to cross-validate the factor structure. It also would be of interest to cross-validate the different international BAS samples and to calculate structural equivalence and invariance. It should also be analysed whether the factors should be correlated or not. It would be worthwhile to further investigate the construct validity and sensitivity to change of the BAS, while also including culture-sensitive analyses of how burden is understood in different cultures and socio-economic contexts. The BAS should be used in representative samples of relatives with the aim to create norms for different populations. A copy of the German BAS is available from the first author.

## Abbreviations

BAS: Burden Assessment Scale
BSI: Brief Symptom Inventory
BSI-GSI: Brief Symptom Inventory, Global Severity Index
CFA: confirmatory factor analysis
CI: confidence interval
DMH&H: New Jersey Division of Mental Health and Hospitals study
EXIS: Experience in Social Systems Questionnaire
EXIS.pers: Experience in Social Systems Questionnaire, personal social systems
EXIS.org: Experience in Social Systems Questionnaire, organizational social systems
